# Occupational exposure to blood and body fluids and anxiety levels among healthcare workers

**DOI:** 10.1192/j.eurpsy.2026.11656

**Published:** 2026-07-31

**Authors:** H. Daoud, I. Sellami, A. Feki, M. A. Ghrab, M. Hajjaji, M. L. Masmoudi, K. Jmal Hammami

**Affiliations:** 1 Department of occupational medicine, Hedi Chaker Hospital; 2 LR/18/ES-28, Sfax university; 3 Department of rheumatology, Hedi Chaker Hospital, Sfax, Tunisia

## Abstract

**Introduction:**

Healthcare workers (HCWs) face daily risks of occupational exposure to blood and body fluids (OEBBF). These incidents are often associated with organizational and psychosocial factors, which may heighten anxiety and impair safe professional practices.

**Objectives:**

This study aimed to assess anxiety levels of HCWs with recent OEBBF.

**Methods:**

We conducted a cross-sectional study including all newly reported cases of OEBBF consulting the occupational medecine department during the period between January 2023 and December 2023. Data collection included sociodemographic, occupational characteristics and exposure-related details. Anxiety symptoms were assessed using the Generalized Anxiety Disorder 7-item (GAD-7) scale, a validated screening tool for detecting and grading anxiety severity.

**Results:**

Of a total of 201 reported OEBBF, 83.1% were needle-stick injuries, 7.5% were cuts from sharp objects, and 9.5% reported biological fluid splash. Injured women represented 86.8%. Mean age average was 25.38 ± 4.94 years. Medical interns represented the largest group with 48.8%. The mean GAD-7 score among HCWs was 4.73 ± 3.60. Overall, 22 participants (11.0%) presented clinically significant anxiety (GAD-7 ≥10).

Bivariate analysis showed no significant association between GAD-7 scores and age, sex, type of exposure, or category of HCWs.

**Conclusions:**

Occupational exposure to blood and body fluids (OEBBF) is common among healthcare workers (HCWs). High levels of anxiety are frequently observed in individuals who have recently experienced OEBBF, and anxiety is associated with such exposures, highlighting the importance of psychological support and targeted preventive strategies.

**Disclosure of Interest:**

None Declared

